# Protein-Directed Dynamic Combinatorial Chemistry: A Guide to Protein Ligand and Inhibitor Discovery

**DOI:** 10.3390/molecules21070910

**Published:** 2016-07-16

**Authors:** Renjie Huang, Ivanhoe K. H. Leung

**Affiliations:** School of Chemical Sciences, The University of Auckland, Private Bag 92019, Victoria Street West, Auckland 1142, New Zealand; rhua571@aucklanduni.ac.nz

**Keywords:** dynamic combinatorial chemistry, enzyme inhibition, ligand screening, drug discovery, ligand binding, reversible reaction, biophysical techniques

## Abstract

Protein-directed dynamic combinatorial chemistry is an emerging technique for efficient discovery of novel chemical structures for binding to a target protein. Typically, this method relies on a library of small molecules that react reversibly with each other to generate a combinatorial library. The components in the combinatorial library are at equilibrium with each other under thermodynamic control. When a protein is added to the equilibrium mixture, and if the protein interacts with any components of the combinatorial library, the position of the equilibrium will shift and those components that interact with the protein will be amplified, which can then be identified by a suitable biophysical technique. Such information is useful as a starting point to guide further organic synthesis of novel protein ligands and enzyme inhibitors. This review uses literature examples to discuss the practicalities of applying this method to inhibitor discovery, in particular, the set-up of the combinatorial library, the reversible reactions that may be employed, and the choice of detection methods to screen protein ligands from a mixture of reversibly forming molecules.

## 1. Introduction

The development of novel chemical agents to inhibit the catalytic activity of disease-causing enzymes is a major theme of medicinal chemistry research and drug discovery programmes. Currently, high-throughput screening (HTS) is one of the most utilised methods—particularly in the pharmaceutical industry—to identify chemical structures that are important for binding and inhibiting target enzymes, and act as a starting point for subsequent organic synthesis and structural-activity relationship studies [1,2,3,4]. Whilst HTS has proven to be an invaluable tool in inhibitor and drug discovery, it can be expensive, time consuming and—depending on the composition of the screening library—the screen may only cover a limited chemical space, thus potentially limiting the number of unique chemical scaffolds that can be identified from HTS [5,6,7,8].

There is a paradigm shift in the drug discovery community to move away from screening large libraries (e.g., hundreds of thousands of compounds) to the use of alternative screening approaches and focus on much smaller chemical libraries (e.g., a few thousand compounds maximum) [9]. The protein-directed dynamic combinatorial chemistry (DCC) method [10,11,12,13,14,15,16,17,18] and the related fragment-based screening method [19,20,21,22,23] are two examples of these alternative screening approaches, which, when compared to HTS, are relatively inexpensive and allow the exploration of large chemical space in a more efficient manner.

The concept of protein-directed DCC was coined in the late 1990s by the supramolecular chemistry community [24], and since then, there have been rapid developments in the field. Protein-directed DCC relies on a library of small molecules that can react reversibly with each other to generate chemical diversity [10,11,12,13,14,15,16,17,18]. Early publications have focussed on proof-of-principle studies, which included investigating the types of reversible chemical reactions that are compatible with protein-directed DCC, and the range of biophysical techniques that are suitable for the detection of protein binders from a dynamic and complex mixture. The technology has matured over the last decade, and there are now numerous examples of successful applications of protein-directed DCC to discover novel enzyme inhibitors that are of medicinal interest, in particular from academic research laboratories. A review article published last year has made an excellent summary of these examples [18].

The aim of this review is to use literature examples to discuss the practical application of protein-directed DCC to discover protein ligands and enzyme inhibitors. It begins with a brief introduction about the concept of protein-directed DCC, which is then followed by a more in-depth discussion about the factors that one should consider when setting up a protein-directed DCC screening project.

## 2. The Protein-Directed DCC Method

### 2.1. General Concept

**Figure 1:**
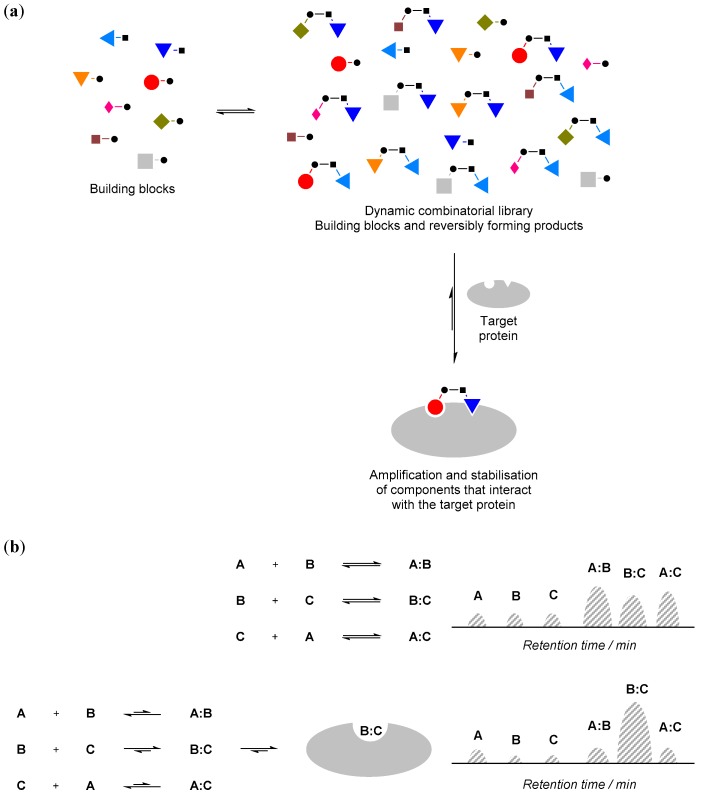
(**a**) The principle of protein-directed dynamic combinatorial (DCC). • and ▪ denote functional groups for reversible reactions; (**b**) If a component of the dynamic combinatorial library (DCL) interacts with the protein (e.g., component **B**:**C**), its concentration will increase and the concentration of the other components will decrease. Such changes can be monitored by an appropriate biophysical technique such as high performance liquid chromatography (HPLC; e.g., schematic illustration of HPLC traces is shown above).

The basic make-up of a protein-compatible combinatorial library typically involves a mixture of small and simple molecules that can react reversibly with each other in aqueous solution and at physiologically-relevant pH. These molecules are often referred to as ‘building blocks’. All of these small molecule building blocks and their reaction products are at equilibrium with each other in the mixture under thermodynamic control, and their relative distribution in the mixture reflects their respective thermodynamic stability within the equilibrium (Figure 1a).

Such a combinatorial library can be highly dynamic in nature. For instance, when an ‘external influence’, such as a protein, is added to the combinatorial library, and if the protein interacts with (i.e., binds) some components of the mixture, according to Le Chatelier’s principle, the equilibrium position will shift and those components that interact with the protein will be amplified (Figure 1b). These hits can then be used to guide organic synthesis to obtain ‘stable analogue’ inhibitors for further testing. The equilibrium mixture of the reversibly reacting building blocks is called a ‘dynamic combinatorial library (DCL)’, and the external influence (protein) is often referred to as a ‘template’.

Practically, the design of any protein-directed DCC experiments should include four components: (1) a protein template; (2) a library of building blocks; (3) a reversible reaction; and (4) analytical method(s).

### 2.2. Composition of a Dynamic Combinatorial Library

Library design is of utmost importance in protein-directed DCC. Unlike HTS, whose libraries contain a large number of structurally diverse compounds, DCLs tend to be more focussed and are usually guided by protein structural information. Typically, the design of a DCL may begin with a so-called ‘scaffold molecule’. A scaffold ligand is a ligand that is designed to bind the target protein, and at the same time contains a functional group for reversible reaction with other components of the DCL. The design of the scaffold molecule may be based on an existing binder, by analyses of the target protein structure, or by molecular modelling. The reversible reaction functional group can be placed in a way that it is pointing out towards a secondary binding pocket where additional interactions are desired.

The reason that protein-directed DCC is rarely applied in a completely random manner is likely due to practicality. As each building block in a DCL should contain at least one functional group for the reversible reaction, it is expensive and impractical to synthesise thousands of custom-made building blocks to generate a truly random DCL. The use of a scaffold ligand ensures that at least one molecule in the DCL will bind the target protein. In order to generate chemical diversity within the DCL, the scaffold ligand is complemented with a library of small molecules, each of them containing a functional group to react reversibly with the scaffold ligand, and different structures to cover different geometrical and functional spaces.

The concept of using a scaffold ligand to explore a secondary binding pocket was demonstrated by Demetriades et al. in 2012 [25]. Prolyl hydroxylase domain 2 (PHD2) is a Fe(II) metalloenzyme that contains two neighbouring binding pockets: one binds the co-substrate 2-oxoglutarate (2OG) via the metal ion Fe(II), and the other binds its protein substrate hypoxia inducible factor (HIF). Many PHD2 inhibitors are designed to compete against 2OG for binding to the ferrous iron in the co-substrate binding site, and it is postulated that selectivity and enhanced inhibition potency may be achieved by extending such inhibitors into the substrate binding pocket. The reversible boronate ester formation reaction, which involves boronic acids and diols, was used to generate the DCL.

**Figure 2:**
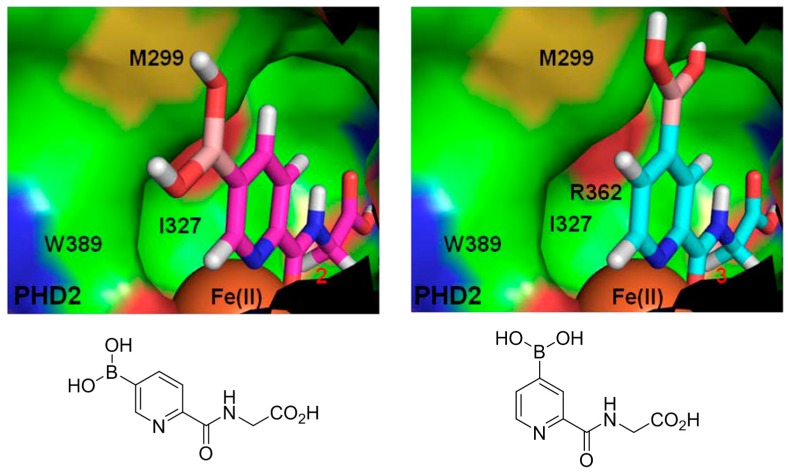
Two scaffold ligands, both contain a boronic acid moiety, were used to explore the geometry between the co-substrate and substrate binding sites of prolyl hydroxylase domain 2 (PHD2). Whilst both boronic acids bind to PHD2 via the active site Fe(II), only the scaffold ligand with the boronic acid moiety at 5-position is able to form boronate esters with diols in the presence of PHD2, as the boronic moiety is pointing towards the substrate binding pocket (modified and reprinted with permission from [25]).

The authors first designed a scaffold ligand, which contains a pyridine nitrogen and a carboxylic acid group to chelate the PHD2 active site metal ion, based on existing PHD2 inhibitors that compete against 2OG. The next step involved the introduction of the boronic acid moiety onto the scaffold ligand so that it can form boronate esters with diols in the DCL to explore the PHD2 substrate binding pocket. Two scaffold ligands with the boronic acid moiety substituted at either the 4 or 5 position of the pyridine ring were synthesised. Whilst both scaffold ligands bind to PHD2 on their own, the authors showed that only the scaffold ligand with the boronic acid moiety pointing towards the substrate binding pocket is able to form boronate esters with diols in the presence of PHD2 (Figure 2 and Table 1). Using this scaffold ligand, a series of boronate esters that contain hydrophobic aromatic ring(s) were identified with low μM or nM binding constants (*K*_D_) by mass spectrometry and nuclear magnetic resonance (NMR) spectroscopy. Based on these information, several ‘stable analogues’, in which the boronate ester bond of the boronate ester is replaced by a C-C bond, were synthesised (Table 1). Further optimisation based on protein crystallography data led to the discovery of several novel inhibitors with nanomolar *K*_D_ and inhibition constant (IC_50_) to the enzyme (Table 1).

In addition to PHD2, the use of Fe(II) chelator as a scaffold ligand was also applied to a number of 2OG-dependent oxygenases including Jumonji domain 2 proteins (JMJD2) [26], which are a subfamily of histone demethylases, and AlkB [27], which is a deoxyribonucleic acid (DNA) demethylase. Similar to PHD2, the scaffold ligands were designed based on generic 2OG oxygenase inhibitors that compete against 2OG for chelation to the active site Fe(II). However, by placing the substituent that contains the functional group responsible for reversible DCC reactions at different positions on the scaffold ligand, the authors were able to obtain selectivity and potency amongst these members of the 2OG-dependent oxygenase superfamily, which share similar structural features at the 2OG co-substrate binding site but different substrate binding pocket.

In another example, Mondal et al. applied protein-directed DCC to develop inhibitors for endothiapepsin, which is a pepsin-like aspartic protease [28]. The active site of endothiapepsin contains a catalytic dyad (Asp35 and Asp219). These two amino acids cleave the peptide bond of the enzyme’s peptidyl substrate with the help of a bound water molecule. Endothiapepsin is often used as a model enzyme for mechanistic studies and for the development of other protease inhibitors such as those that target renin and β-secretase. In this example, the reversible acylhydrazone formation reaction, which involves hydrazines and aldehydes, was used.

The authors started by studying the crystal structures of endothiapepsin. They focussed on two different structures from the protein data bank. Both endothiapepsin structures contain a bound small fragment molecule. However, the binding modes of these two fragments are different. One of the fragments is bound to the protein with a crystallographically localised water molecule, whilst the other is bound directly with the catalytic dyad via hydrogen bond.

**Figure 3:**
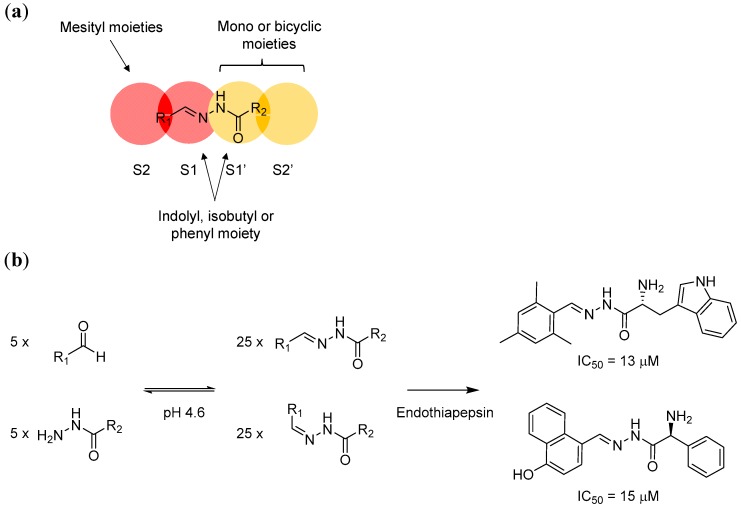
(**a**) In silico modelling of acylhydrazone has led to the identification of potential structural moieties that fit into the endothiapepsin S1, S2, S1’ and S2’ sites; (**b**) The application of protein-directed DCC using hydrazone formation reaction has led to the discovery of potent endothiapepsin inhibitors. The DCL was designed based on the information obtained from in silico modelling. The two most potent hits from the series in turn confirm the modelling results.

Using these two protein structures (but without considering the fragments), the authors conducted de novo in silico inhibitor design and modelled the binding of potential acylhydrazone products with and without the localised water molecule inside the binding site. The modelling studies suggest that the S1’ and S2’ pockets, which are carboxy-terminal (C-terminal) to the scissile bond, can accommodate both mono- and bicyclic aromatic moieties. The hydrophobic S2 pocket, which is amino-terminal (N-terminal) to the scissile bond, is best occupied by a mesityl substituent, whilst the S1 and S1’ pockets can host an indolyl, isobutyl, or phenyl moiety (Figure 3a). Based on these models, five hydrazines and five aldehydes (forming 50 different acylhydrazones including *E*/*Z* isomers) were screened initially by NMR spectroscopy (which cannot distinguish *E*/*Z* isomers). The active hydrazones were then crystallised with endothiapepsin and studied by protein X-ray crystallography. In agreement with the modelling studies, endothiapepsin selectively binds the *E* isomer from the mixture of *E*/*Z* isomers (Figure 3b). Overall, this example highlights the power of combining in silico modelling and protein-directed DCC to generate novel protein ligands or inhibitors.

**Figure 4:**
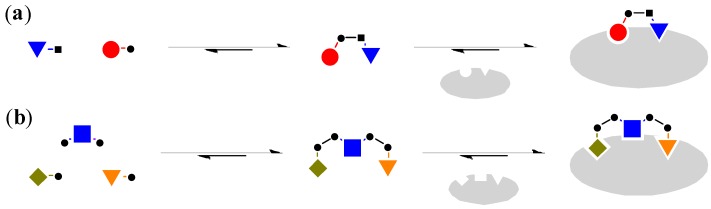
(**a**) Mono-functionalised building blocks to generate dimeric molecules [29]; (**b**) The use of bi-functionalised linker and mono-functionalised head groups to generate trimeric molecules [30].

Although the most common way to generate DCL, such as the examples mentioned above, involves the use of mono-functionalised building blocks to generate dimeric molecules [29], it is also possible to use bi-functionalised linker molecules and mono-functionalised head groups for the combinatorial library (Figure 4) [30].

The use of a bi-functionalised linker molecule was demonstrated by Cancilla et al. in the quest to discover Aurora A kinase inhibitors [31]. Many kinase inhibitors to date are designed to occupy the adenosine triphosphate (ATP) binding pocket. However, such inhibitors often lack selectivity. In the case of Aurora A, it is postulated that selectivity may be achieved by targeting a second adaptive region, which is located a few angstroms away from the ATP binding site. However, targeting the adaptive site poses a different challenge as it requires the binding of ATP (or an ATP mimic) at the ATP binding site to trigger protein conformational change. In order to achieve that, the authors utilised a technique called ‘tethering’ [32], in which the scaffold molecule is covalently linked to the protein, in tandem with protein-directed DCC to probe the adaptive binding site. The reversible disulphide bond formation reaction using thiols were used as the reversible reaction.

**Figure 5:**
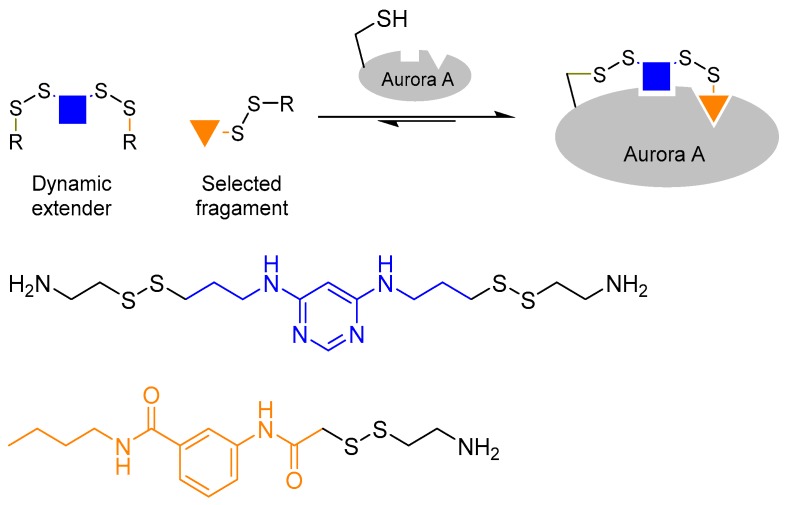
The combined use of tethering and protein-dynamic combinatorial chemistry to discover Aurora A kinase inhibitors [31]. The authors tethered the scaffold ligand onto Aurora A to access the adaptive binding pocket in order to develop selective kinase inhibitors.

Firstly, the authors chose diaminopyrimidine, a known structure that binds kinase ATP binding sites, as the core of the scaffold ligand. The diaminopyrimidine core was then bi-functionalised with two thiol moieties. Next, the authors introduced a cysteine residue (which contains a thiol side-chain) into the Aurora A ATP binding site by mutagenesis. This allows one of the thiol moieties of the diaminopyrimidine scaffold ligand to react with the introduced cysteine residue inside the ATP binding site once the scaffold ligand is bound to the kinase. The tethering of the scaffold ligand to the protein allows access to the adaptive site, and enables the scaffold ligand to act as a dynamic extender to explore the adaptive binding pocket by reacting reversibly with the other thiol-containing building blocks in the DCL (Figure 5). This strategy has successfully led to the discovery of new Aurora A inhibitors that bind the kinase adaptive pocket, which will otherwise be difficult to achieve.

The application of protein-directed DCC is not limited to enzyme inhibitors. It can also be used to design protein binders for biochemical applications. For example, posttranslational modifications of histone proteins such as lysine (de)methylation may affect chromosome structure and function and therefore play an important role in both development and disease. However, the detection of different lysine methylation states in vivo is not a trivial task, and the lack of binders that selectively recognise different lysine methylation states hinders our understanding of the histone code.

**Figure 6:**
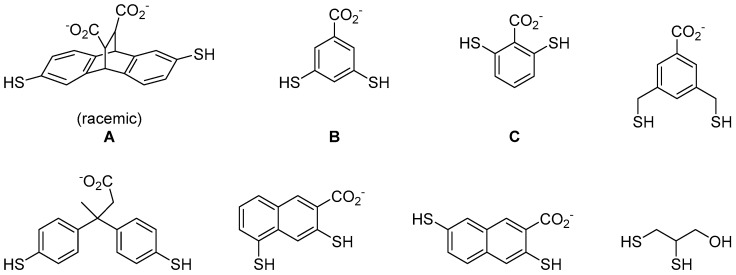
The use of dithiol aromatic building blocks to discover compounds that recognise trimethyllysine on histone peptides. *rac*-A_2_B, *meso*-A_2_B, A_2_C and AC_3_ were amplified when screened against a dipeptide containing trimethyllysine [33].

To try to solve this problem, Ingerman et al. applied thiol-disulphide exchange reaction to develop compounds that can selectively recognise histones with trimethyllsines [33]. He used eight different dithiol aromatic building blocks to construct a DCL (Figure 6). The use of aromatic compounds is to facilitate cation-π interactions with the trimethylammonium motif on trimethyllysines. These building blocks also contain carboxylic acid group(s) to promote water solubility. In the presence of a dipeptide Ac-KMe_3_-G-NH_2_ template, three (out of the eight) building blocks were identified as important for recognising the trimethyllysine moiety (Figure 6). In order to confirm the selectivity of these conjugates, the authors then conducted a second screen with dipeptides of the same amino acid sequence but this time the trimethyllysine residue was substituted by lysines of different methylation states. Finally, the binders were isolated by high performance liquid chromatography (HPLC) and their binding affinity to H3 K9 peptides were measured (Table 2). The authors showed that DCC is a promising technique to the development of protein binders for the detection of posttranslational modification on proteins in vivo.

Finally, although most applications of protein-directed DCC to date involve the use of small molecule building blocks, it is possible to use macromolecular (such as peptidyl) building blocks to probe protein-protein interactions. This concept is exemplified by a recent study conducted by Eisenberg et al. [34].

Repeat proteins are found in almost all cellular systems. They function as triggers and binders in diverse molecular recognition processes. For example, in eukaryotes, heat shock protein 70 (HSP70) and heat shock protein 90 (HSP90) require the help of tetratricopeptide repeat (TPR) domain-containing co-chaperones in order to perform many of their biological functions. It is postulated that synthetic repeat proteins may be used as ‘universal protein binders’ in a similar manner to antibodies for biochemical applications. However, total synthesis of large repeat proteins is difficult, and this limits the research and application of repeat proteins in biochemistry.

**Figure 7:**
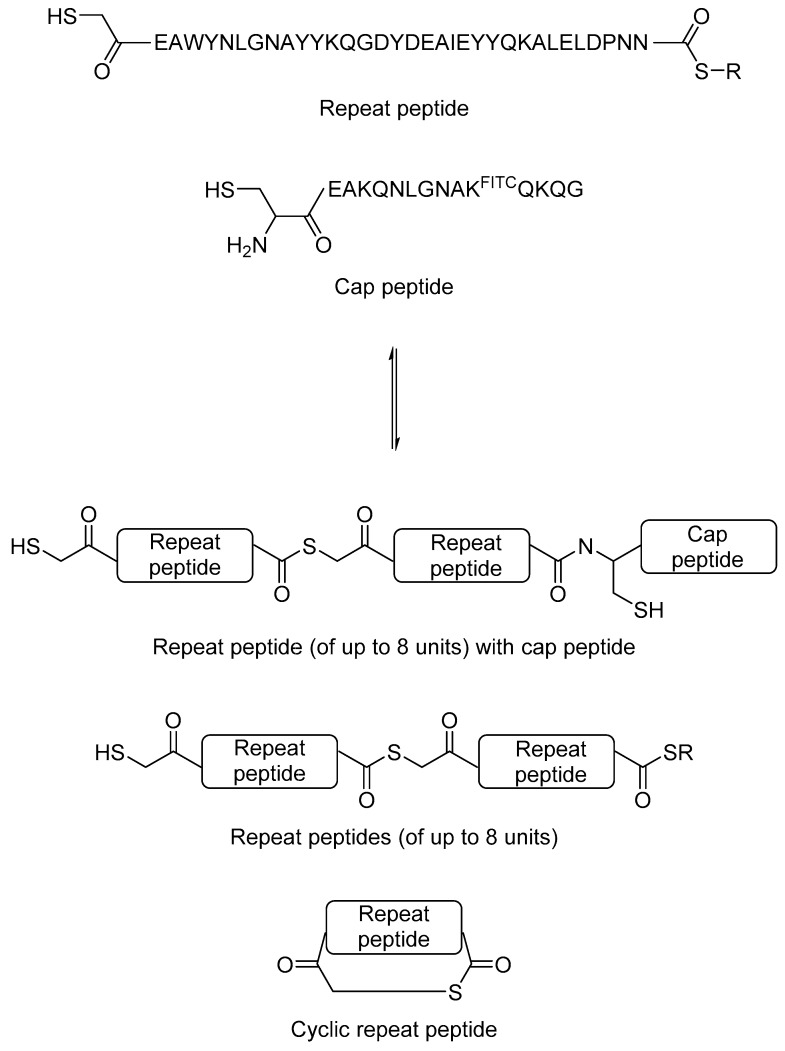
The use of thiol-thioester exchange reaction to generate dynamic combinatorial library of peptides and proteins [34].

Although total synthesis of large proteins is not trivial, synthesis of short peptides using solid phase peptide synthesis is now a routine process. Eisenberg et al. hypothesised that it may be possible to generate novel repeat proteins by protein-directed DCC using peptides as building blocks. As a proof-of-principle study, the authors used a 34-amino acid TPR repeating peptide unit as the building block. The reversible thiol-thioester exchange reaction was used. The authors modified the peptide at the N-terminal to a thiol and at the C-terminal to a thioester. A 15-amino acid C-terminal TPR peptide was used as the C-terminal cap, which contains a fluorescein isothiocyanate (FITC)-labelled lysine to aid detection (Figure 7). The mixture was incubated at a 3:1 repeat peptide to cap peptide ratio in buffer at neutral pH, in which the mixture reached equilibrium after 1–2 days. Multiple products, including a cyclic peptide product from the TPR repeat peptide, and combinatorial proteins of up to eight repeating units were observed. When HSP90 and HSP70 peptides were added to the equilibrium mixture, those combinatorial proteins that interact best with the heat shock protein peptides were amplified, which was then quantified by mass spectrometry. This study showed that protein-directed DCC studies are not limited only to small organic molecules, and that it is possible to generate complex dynamic library of proteins, which opens up the opportunity to de novo protein design in the future.

### 2.3. Reversible Reactions and Reaction Conditions for Protein-Directed DCC

Various dynamic reversible reactions have been proposed and applied in protein-directed DCC, which can be divided into three main types. These include addition-elimination reactions at carbonyl groups (such as imine and hydrazone formation) [24,35,36,37,38,39,40,41,42,43,44,45,46,47,48,49], thiol exchange reactions (such as thiol-disulphide exchange [50,51,52,53,54,55,56,57], thiol-enone reaction [58] and the hemithioacetal reaction) [59,60], and boronate ester formation reaction (Table 3) [25,61,62]. Diselenide exchange and selenenylsulphide exchange reactions have also been shown to react reversibly in water at neutral pH [63,64], although to date there are no examples of inhibitor discovery via protein-directed DCC using these reactions.

Organic-based reactions including alkene cross metathesis and oxime formation have also been applied to protein-directed DCC via a so-called pre-equilibrated approach, in which the combinatorial library is initially generated in organic solvent or organic solvent/water mixture, which is then diluted into aqueous buffer that contains the protein target [65,66]. Although the dynamicity of such organic-based libraries is lost once the mixture is diluted in aqueous solution, the pre-equilibrated approach reduces the need for purification and therefore enables the screening of inhibitors to become a much more efficient process.

The selection of an appropriate reversible reaction for protein-directed DCC is not trivial. Ideally the reaction must occur in aqueous solution at a biologically relevant pH and temperature. The reaction must be compatible with proteins (i.e., it does not denature or react with the protein). The equilibrium should be able to establish (and re-establish) within a reasonably short timescale to allow efficient screening. 

**Figure 8:**
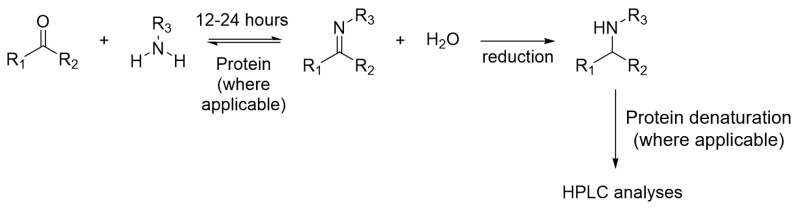
The reduction of imine DCL for HPLC analyses.

Imine formation is one of the first reversible reactions demonstrated for protein-directed DCC. The reaction can occur at neutral pH and the equilibrium can establish within 12 and 24 h. Imine formation DCL is well established and most literature examples couple imine DCL with HPLC for detection, in which imines are reduced chemically by the use of sodium cyanoborohydride to form a stable analogue before analyses by HPLC (Figure 8).

Similar to imine formation, thiol-disulphide exchange reaction is also amongst some of the first reversible reactions demonstrated for protein-directed DCC, as they typically occur at neutral pH. However, such libraries often require long incubation time to reach equilibrium, as evident by earlier work conducted by Ramström and Lehn [50] and Milanesi et al. [51], in which their DCLs were left for two weeks and four days for equilibration, respectively. An improvement to the applicability of thiol-disulphide exchange in protein-directed DCC was demonstrated by Scott et al. [53]. In this work, 5′-deoxy-5′-thioadenosine was used as a scaffold ligand together with a small library of thiols to discover *Mycobacterium tuberculosis* pantothenate synthetase inhibitors using thiol-disulphide exchange. The thiols were present at equimolar concentrations using a glutathione redox buffer at pH 8.5 under inert atmosphere. The glutathione redox buffer promoted disulphide exchange and the DCL reached equilibrium in less than 24 h (both in the presence and absence of the protein). The reaction was then quenched (and if applicable, the protein precipitated) by the addition of strong acids. Finally, the quenched mixture was analysed by HPLC and the amplified binders were identified by HPLC peak height. This protocol by Scott et al. speeded up the equilibration time and enhanced the practicality of using thiol-disulphide exchange to discover protein inhibitors. In additional to using a glutathione redox buffer, selenols and diselenides may also be used to speed up disulphide exchange reactions [67,68]. For example, Rasmussen et al. found that 3,5-dimercaptobenzoic acids, which may react with each other via disulphide formation (to form macrocycles) in water, took seven days to reach equilibrium at pH 7.0. This was reduced to three days by the use of 1 mol % diselenide, and to two days by the use of 10 mol % diselenide [64]. Although the use of diselenides as catalysts has not been applied in protein-directed DCC, the study opened up new potentials to thiol-disulphide DCC in a high-throughput manner.

Another common reversible reaction in protein-directed DCC is the addition-elimination reaction at carbonyl groups such as the hydrazone formation reaction. For example, Ramström et al. applied reversible hydrazone formation to probe the binding site of plant lectin Concanavalin A [45]. Hydrazone exchange reaction typically takes place at acidic pH (pH 3–7), which gets slower as pH increases. The dynamicity of the reaction is essentially stopped at basic pH (above 7.5). In order to generate the combinatorial library, the authors pre-equilibrated the building blocks at pH 4 overnight, which was then diluted to pH 7 for enzymatic assays as a pseudostatic library.

This variability in the dynamicity of hydrazone formation with pH may be advantageous for screening in certain circumstances as the pseudostatic library is less complex than a dynamic library. Sindelar et al. applied hydrazone formation reaction to screen for γ-aminobutyric acid (GABA) transporter 1 (GAT1) inhibitor [46,47]. They have previously designed a liquid chromatography-mass spectrometry (LC-MS)-based competition assay to monitor the percentage displacement of a known binder (reporter) from GAT1 to screen for GAT1 binders. As a dynamic library may complicate LC-MS observations through changes in component concentrations within the equilibrium mixture, the authors used the pseudostatic hydrazone library as an advantage to aid LC-MS detection. Sets of aldehyde compounds with varying structures were reacted with an excess of the hydrazine compound for four hours at 37 °C at neutral or slightly acidic pH. Using this approach, the authors discovered a hydrazone that binds to GAT1 with low nanomolar affinity. Using this information, the authors successfully synthesised a stable analogue inhibitor by replacing the hydrozone moiety with an alkene whilst retaining similar affinity. This example illustrates the importance of choosing the right reversible reaction that is compatible to the detection technique. 

In contrast, boronate ester formation reaction, another commonly used reversible reactions, is at its most dynamic when the pH of the solution is similar to the p*K*_a_ of the boronic acid. In such cases, the equilibrium can re-establish within minutes after it has been perturbed [25,62]. Typically, p*K*_a_ values of arylboronic acids are around 8. This property makes arylboronic acids suitable for reversible reactions at neutral pH. However, the p*K*_a_ values for alkylboronic acids are higher (~10–12). Alkylboronic acids are therefore not suitable for reversible reactions at neutral pH. This is illustrated by Leung et al. in a proof-of-principle study using boronate ester formation to enhance the inhibition of serine protease α-chymotrypsin. The authors showed that at pH 5.8, ethylboronic acid does not form boronate ester with d-fructose (a sugar diol) even at 6x molar excess. They also showed that, unlike other arylboronic acids, 4-hydroxylphenylboronic acid, which contains an electron donating hydroxyl group at the 4-position of the aromatic ring, does not form boronate ester with d-fructose at pH 5.8. Measurement of p*K*_a_ showed that 4-hydroxylphenylboronic acid has a p*K*_a_ of 8.9, presumably due to the electron donating hydroxyl group and resonance effect onto the boron atom. When using boronate ester formation reaction, it is therefore important to measure the p*K*_a_ of the boronic acid and choose a pH that is suitable for both the reversible reaction and compatible with the target protein system.

Finally, Kern and Wanner demonstrated the application of the pre-equilibrated oxime formation approach to screen for neuronal GABA transporter GAT1 inhibitors [66]. Oximes were generated by stirring aldehyde and hydroxylamine in a mixture of DMSO and water in phosphate buffer at 37 °C for 20 h, which was then diluted into aqueous buffer for testing (so that the final DMSO concentration did not exceed 10 μM). Oxime is much more stable against hydrolysis than hydrazone so effectively they remain static and stable in aqueous solution. In such cases, a deconvolution approach can be applied to ensure that the strong binders can be identified.

### 2.4. Protein Template

The role of the protein in protein-directed DCC is to select the best binder from the DCL by changing the equilibrium position of the mixture so that the component(s) that bind best with the protein will increase in concentration. An important criteria to consider is the amount of protein that is required to change the equilibrium of the DCL. For example, Zameo et al. applied imine formation using aldehydes and amines to discover inhibitors for hen egg-white lysozyme (HEWL) [38]. Five hundred micromolar of HEWL (1 equivalent to aldehyde and amine concentrations) was required to stabilise and amplify the best binders, which was then detected by HPLC. In another example, Leung et al. applied boronate ester formation with boronic acids and diols to probe the binding to α-chymotrypsin [62]. Two millimolar of α-chymotrypsin (1 equivalent to the boronic acid concentration) was required as the detection technique (^11^B-NMR) was relatively insensitive. The concentration of proteins required for protein-directed DCC is therefore significantly higher than those conditions that are commonly used in fragment-based drug discovery (low μM to nM concentrations) and this could be a limiting factor if the target enzyme is difficult or expensive to produce.

It is worth noting that the protein concentration required in pre-equilibrated DCL is often low (μM to nM) [35,37,42,48,58,65]. This is because the combinatorial library is no longer dynamic once it is diluted with proteins. Whilst this may be advantageous if the target protein is a limiting factor, such an approach may bias towards detecting the major components that are formed in the pre-equilibrated library rather than the best binder.

### 2.5. Analytical Techniques

Although multiple biophysical techniques have been applied to detect the binders from a DCL, it is still a challenge to choose and apply the right analytical technique due to the dynamic nature of protein-directed DCC. Not only does the technique need to be able to detect protein ligands from a mixture of reversibly reacting molecules, it also needs to be sensitive enough to detect changes in equilibrium and concentrations. Three main types of analytical techniques have been used, including HPLC [24,36,37,38,39,40,41,42,46,47,50,51,52,53,58], native non-denaturing mass spectrometry [26,27,48,56,57], and ligand-observe NMR spectroscopy [53,59,60,61,62]. HPLC and mass spectrometry are particularly useful in protein-directed DCC because they allow the screening of the best binders and the quantification of their relative concentrations from the dynamic mixture. Ligand-observed NMR spectroscopy has also been applied, in particular with the pre-equilibrated approach [53,59,60]. However, such an approach is often complicated by ligand exchange dynamics, as observed by Caraballo et al., who applied a reversible hemithioacetal system with thiol and aldehyde to discover β-galactosidase inhibitors [59,60]. The exchange of thiol and aldehyde at neutral pH is fast on the NMR timescale and as a result their resonances are broadened, which makes them difficult to observe. It is also worth noting that nuclear Overhauser effect (NOE)-based ligand-observe NMR techniques such as saturation transfer difference (STD) and water ligand-observed via gradient spectroscopy (waterLOGSY) are often tuned to detect weak binders with *K*_D_ in the range between μM and mM, so strong binders may not be detected thus leading to false negative results. In addition, false positive results due to non-specific binding are also possible, in particular with a library rich in aromatic and heterocyclic compounds, and in such cases, a complementary assay or technique is needed to confirm the NMR results [59,60]. X-ray crystallography [43] and biological assays have also been applied [35,42,45,60], although the former depends on experimental conditions for crystallisation, and for the latter a deconvoluted approach is required to identify the active compound from the mixture.

Whilst traditional biophysical approaches are well-adapted to screen small to medium size DCL, the identification of the amplified compounds for large DCL remains one of the biggest challenges. In a recent example, Reddavide et al*.* conducted a proof-of-principle study to combine protein-directed DCC that generates chemical diversity with DNA-encoding technology [69], which allows efficient screening of potentially hundreds of thousands of compounds [70,71]. The authors used streptavidin, which is a homotetrameric bacterial protein that binds iminobiotin, as the model protein system. Due to its homotetrameric structure, streptavidin may bind more than one iminobiotin molecules, thus making it a suitable system to illustrate the DCC principle. The authors used DNA-coupled iminobiotin with streptavidin as binder together with unmodified DNA strands as non-binders. Different numbers of hybridisation base pairs ranging from 4 to 21 were investigated. The authors found that if the number of hybridisation base pairs was too large (e.g., 21), the system formed stable DNA and as a result the system became static and no enhancement could be observed in the presence of the protein. Similarly, no enhancements were observed if the number of hybridisation base pairs was too small (e.g., 4 or 5), presumably because the DNA is unstable as there are not enough hydrogen bond interactions. The authors found that if the monomer has high affinity to the protein, the optimal length of base pairs should be 6 to 8, whilst if the monomer has low affinity to the protein, a longer DNA should be used. Although this technology is still in its infancy, it opens up the possibility to screen large DCLs in the future with further method development.

## 3. Summary and Outlook

Protein-directed DCC allows one to access molecular diversity for inhibitor discovery and to conduct structural-activity relationship studies without the need of comprehensive organic synthesis of different structural analogues. To date, most applications of protein-directed DCC can be classified as ‘hit-to-lead’-type projects as they always begin with a scaffold ligand which is designed to bind the target protein, and the method is applied to improve the binding affinity, inhibition potency, and/or selectivity of the ligand towards the target protein. However, the potential of protein-directed DCC is not limited to enzyme inhibitors, and there are interesting recent developments of using protein-directed DCC to design structures that recognise protein posttranslational modifications and peptidyl-based protein ligands. The dynamic nature of this method also possess many challenges for its applications, and attention must be paid to select a suitable reversible reaction that is compatible with the target protein whilst balancing the different factors affecting the dynamicity of the system including pH, incubation time, reactivity of the reversible reaction, and the concentrations of the small molecule building blocks and proteins. Several biophysical techniques have been applied to identify protein ligands from a DCL, and HPLC and mass spectrometry have emerged as the method-of-choice for many protein-directed DCC studies. In addition, novel technologies such as those that couple DCC with DNA encoded library are in development, which may allow the screening of much larger DCL in the future.

Over the last 15 years or so the protein-directed DCC technique has gone from the method development stage and the method has now led to the discovery of numerous novel protein ligands and enzyme inhibitors for many different protein systems. Although successful projects so far have been conducted mainly by academic laboratories, we believe the technology is now mature and we envisage the method will carry on to be refined and applied to discover more enzyme inhibitors by both the academia and industrial communities.

## Figures and Tables

**Table 1:** The application of protein-directed dynamic combinatorial chemistry has led to the discovery of novel nanomolar IC_50_ inhibitors for PHD2 [25]. Asterisk (*) denotes the apparent binding constant of the boronate ester species. Inhibition constant (IC_50_) was measured for the ‘stable analogues’ only, in which the boronate ester bond of the conjugate is replaced by a C-C bond.

Compound(s)	*K*_D_/μM	IC_50_/μM
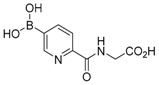 Boronic acid ‘scaffold ligand’	24.8	126
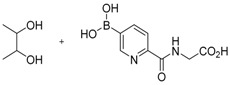 Boronate ester conjugate	18.2 *	N/A
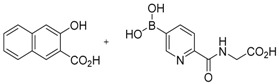 Boronate ester conjugate	3.0 *	N/A
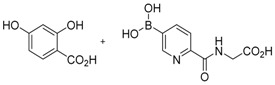 Boronate ester conjugate	0.8 *	N/A
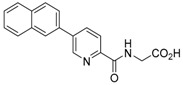 First generation stable analogue	7.0	>500
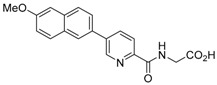 First generation stable analogue	1.6	107
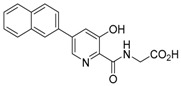 Second generation stable analogue	0.5	0.017
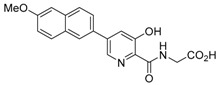 Second generation stable analogue	0.8	0.013

Asterisk (*) denotes the apparent binding constant of the boronate ester species.

**Table 2:** HPLC-purified *rac*-A_2_B, *meso*-A_2_B showed selectivity for H3 K9 Me_3_ peptides [33].

Peptide	*rac*-A_2_B (*K*_D_/μM)	*meso*-A_2_B (*K*_D_/μM)
H3 K9Me_3_	25 ± 3	28 ± 4
H3 K9Me_2_	58 ± 10	73 ± 9
H3 K9 Me	166 ± 50	not determined
H3 K9	>1200	not determined

**Table 3:** Common reversible reactions that are used in protein-directed DCC.

Reaction	Scheme	Notes
Imine formation	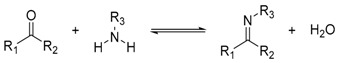	Dynamic at neutral pH Different incubation times have been reported, but typically 12 to 24 h
Hydrazone formation	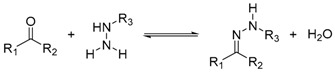	Dynamic at acidic pH Pseudostatic library at neutral pH 4 to 24 h incubation time
Thiol-disulphide exchange	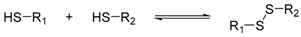	Dynamic at neutral pH Around 24 h incubation time in inert atmosphere in glutathione redox buffer Potential to speed up incubation time by the use of diselenide catalyst Longer incubation time required if exposed to air in normal buffer
Thiol-enone exchange	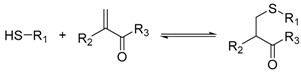	Dynamic at neutral pH Different incubation times have been reported ranging from hours to days
Hemithioacetal reaction	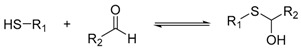	Dynamic at neutral pH Short incubation time (minutes)
Boronate ester formation	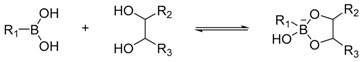	pH similar to p*K*_a_ of boronic acid Works well for arylboronic acids but not for alkylboronic acids at neutral pH Short incubation time (minutes).

## Abbreviations

The following abbreviations are used in this manuscript:

2OG	2-Oxoglutarate
ATP	Adenosine triphosphate
DCC	Dynamic combinatorial chemistry
DCL	Dynamic combinatorial library
DNA	Deoxyribonucleic acid
FITC	Fluorescein isothiocyanate
GABA	γ-Animobytyric acid
GAT1	γ-Animobytyric acid transporter 1
HEWL	Hen egg-white lysozyme
HIF	Hypoxia inducible factor
HPLC	High-performance liquid chromatography
HSP	Heat shock protein
HTS	High-throughput screening
JMJD2	Jumonji domain 2
LC-MS	Liquid chromatography-mass spectrometry
NMR	Nuclear magnetic resonance
NOE	Nuclear Overhauser effect
PHD2	Prolyl hydroxylase domain 2
STD	Saturation transfer difference
TPR	Tetratricopeptide repeat
WaterLOGSY	Water ligand-observed via gradient spectroscopy
